# Self-Healing Hydrogels Fabricated by Introducing Antibacterial Long-Chain Alkyl Quaternary Ammonium Salt into Marine-Derived Polysaccharides for Wound Healing

**DOI:** 10.3390/polym15061467

**Published:** 2023-03-15

**Authors:** Rongkai Li, Qinbing Qi, Chunhua Wang, Guige Hou, Chengbo Li

**Affiliations:** Key Laboratory of Medical Antibacterial Materials of Shandong Province, School of Pharmacy, Binzhou Medical University, Yantai 264003, China

**Keywords:** wound healing, self-healing hydrogels, antibacterial, double cross-linked

## Abstract

The development of hydrogels as wound dressings has gained considerable attention due to their promising ability to promote wound healing. However, in many cases of clinical relevance, repeated bacterial infection, which might obstruct wound healing, usually occurs due to the lack of antibacterial properties of these hydrogels. In this study, we fabricated a new class of self-healing hydrogel with enhanced antibacterial properties based on dodecyl quaternary ammonium salt (Q12)-modified carboxymethyl chitosan (Q12-CMC), aldehyde group- modified sodium alginate (ASA), Fe^3+^ via Schiff bases and coordination bonds (QAF hydrogels). The dynamic Schiff bases and coordination interactions conferred excellent self-healing abilities to the hydrogels, while the incorporation of dodecyl quaternary ammonium salt gave the hydrogels superior antibacterial properties. Additionally, the hydrogels displayed ideal hemocompatibility and cytocompatibility, crucial for wound healing. Our full-thickness skin wound studies demonstrated that QAF hydrogels could result in rapid wound healing with reduced inflammatory response, increased collagen disposition and improved vascularization. We anticipate that the proposed hydrogels, possessing both antibacterial and self-healing properties, will emerge as a highly desirable material for skin wound repair.

## 1. Introduction

The skin serves as the body’s outermost layer to shield the interior organs from exterior hazards and invasions [1]. However, the skin is vulnerable to damage due to injury or illness. In recent decades, dressings have been widely used because they are relatively simple and safe wound treatment options [2,3,4]. Hydrogels stand out among wound dressings due to their alluring abilities which are beneficial for wound healing, such as forming physical barriers to keep the wound away from microbial invasion, and sustaining an ideal moisture environment at the wound site [5,6,7,8]. Unfortunately, traditional hydrogel dressings on the market lack repeated wound closure effects, so they often suffer from poor long-term stability [9]. The need for self-healing materials has driven the development of hydrogels that can autonomously repair their network structure following damage [10,11,12,13]. Self-healing hydrogels are commonly obtained by various mechanisms, including covalent and non-covalent bonds such as coordination bonds [14], Schiff bases [15], Diels-Alder reactions [16] and hydrogen bonds [17]. It should be noted that hydrogels cross-linked by dynamic bonds exhibit self-healing capacities. When hydrogels based on dynamic bonds are destroyed, the internal dynamic cross-linked networks can undergo reversible fracture and reorganization, resetting the hydrogels to their initial state.

Marine-derived polysaccharides are promising biomaterials for the fabrication of hydrogels due to their obvious advantages of colossal storage, non-toxicity and superior biocompatibility [18]. Notably, the backbone of marine-derived polysaccharides contains several hydroxyl, carboxyl and amido groups, which allows tailoring of certain properties of hydrogels to adapt to different requirements by chemical modification and the addition of active substances. Sodium alginate (SA) has emerged as an important marine-derived polymeric biomaterial for biomedical and tissue engineering applications, owing to its significant superiorities in wound debridement, both to maintain a moist wound healing environment and to dissolve necrotic tissue and exudate together [19]. Chitosan (CS) is another marine-derived polysaccharide, and its relatively poor solubility limits its application in wound dressings [20,21]. Carboxymethyl chitosan (CMC) is the carboxymethylated form of chitosan that has better water solubility than chitosan [22]. However, the antibacterial properties of CMC are limited in neutral environments, as its antibacterial action depends on the protonation of amino groups under acidic conditions [23]. Several strategies have been explored to enhance the antibacterial properties of CMC-based wound dressings, such as the incorporation of antibiotics [24] and silver nanoparticles [25]. However, antibiotic overuse can lead to drug resistance of bacteria, and the high cost and biotoxicity of silver nanoparticles make them less appealing. Recently, the use of quaternary ammonium salts (QAS) has gained attention due to their efficient and permanent antibacterial properties [26,27,28,29,30]. The antibacterial activity of QAS with a long-chain alkyl structure is enhanced with the increasing chain length of alkyl groups in the ammonium groups [31,32]. It has been reported that dodecyl quaternary ammonium salt (Q12) is particularly active against microorganisms [33,34,35]. To the best of our knowledge, the use of CMC modified with long-chain alkyl QAS in the preparation of hydrogel wound dressings has not been widely reported.

Given the facts above, we fabricated self-healing hydrogels based on Q12-CMC, ASA and Fe^3+^, formed via Schiff bases and coordination bonds, with the aim of improving wound healing and reducing patient discomfort. Owing to the long chain alkyl QAS, the obtained hydrogels exhibited enhanced antibacterial abilities. In addition, the hydrogels could smartly utilize the double dynamic cross-linked network to obtain self-healing abilities. Notably, Fe^3+^ showed good biocompatibility and hemostatic capacities, making it a good candidate to prepare hydrogels [12]. The morphology, chemical structure, uptake properties, rheological properties, self-healing properties and antibacterial abilities were systematically characterized. In vitro hemocompatibility and cytocompatibility evaluation of QAF hydrogels were tested by the cytotoxic test, hemolysis test and clotting test. Finally, in a full-thickness skin defect model, quantitative statistical analysis of wound contraction and observation of wound healing were employed to investigate the therapeutic efficacy of QAF hydrogel wound dressings. Experimental results showed the proposed hydrogels with outstanding self-healing abilities, antibacterial properties, hemocompatibility and cytocompatibility had promising application prospects in wound healing and preventing bacterial infections of the wounds.

## 2. Materials and Methods

### 2.1. Materials

Carboxymethyl chitosan (Mn = 150,000–800,000, with a degree of deacetylation and degree of carboxymethyl substitution of 90% and 80%) was purchased from Yuanye Bio-Technology Co., Ltd. (Shanghai, China) Yeast powder, trypsin and Sabouraud agar medium were obtained from British OXOID. *N*-dodecylethanolamine, ferric trichloride (FeCl_3_), sodium chloride (NaCl), sodium periodate (NaIO_4_), sodium hydroxide (NaOH) and ethanol were purchased from Sinopharm Chemical Reagent Co., Ltd. (Shanghai, China) Fluorescein diacetate (FDA) and propidium iodide (PI) were provided by Sigma-Aldrich. Dialysis membrane (MWCO = 1000 Da) was obtained from Viskase Cos. Inc. (Chicago, Illinois, USA). Sodium alginate, epichlorohydrin and D_2_O (99.9 atomic % D) were purchased from Shanghai Macleans Biochemical Technology Co., Ltd. (Shanghai, China) Dulbecco’s Modified Eagle Media (DMEM) and Fetal Bovine Serum (FBS) were purchased from Hyclone. All the reagents were used without further purification unless otherwise noted. Cell Counting Kit-8 (CCK-8) was procured from Beijing Solarbio Science & Technology Co., Ltd. (Beijing, China) *S. aureus* (ATCC6538) and *E. coli* (ATCC25922) were purchased from American Type Culture Collection.

### 2.2. Preparation of ASA

To prepare aldehyde-modified sodium alginate (ASA), a 300 mL deionized water solution containing 2 g of SA was prepared and treated with a suitable quantity of sodium periodate (NaIO_4_). The suspension was stirred for 24 h in the dark, and excess ethylene glycol was added to remove unreacted NaIO_4_. After two hours of reaction, 0.3 g NaCl was added. The suspension was dialyzed for 3 d and then freeze-dried. The aldehyde content of ASA was determined to be 4.8 mmol/g using the hydroxylamine hydrochloride method.

### 2.3. Preparation of QAF Hydrogels

Excessive epichlorohydrin (2.5 mol) was added to dodecyl diethanolamine (0.5 mol) and the slurry was stirred at 60 °C for 4 h. An appropriate amount of distilled water was then added to achieve azeotropy with residual epichlorohydrin. Then, the reaction medium was concentrated using a rotary vacuum evaporator at 50 °C and recrystallized to acquire Q12.

Q12 and CMC were mixed in distilled water at a volume ratio of 2:1 and shaken at 85 °C for 24 h, after which the product was precipitated with substantial absolute ethanol. The precipitate was washed three times with anhydrous ethanol, followed by dialysis for 72 h and lyophilization to yield Q12-CMC. 5% (*w*/*v*) Q12-CMC, 5% (*w*/*v*) ASA and 1% (*w*/*v*) FeCl_3_ were mixed to prepare QAF hydrogels. As a control, 5% (*w*/*v*) CMC, 5% (*w*/*v*) ASA and 1% (*w*/*v*) FeCl_3_ were mixed to prepare CAF hydrogels.

### 2.4. Characterizations

The nuclear magnetic resonance (^1^H NMR) spectra of CMC and Q12-CMC were recorded on an AVANCE 600 MHz (Bruker, Billerica, Massachusetts, USA) with D_2_O as a solvent.

Fourier transform infrared (FT-IR) spectra of SA, ASA, CMC, Q12-CMC and QAF hydrogels were obtained using a FTIR spectrometer (Nicolet iS 50, Thermo Fisher Scientific, Waltham, MA, USA) by collecting 32 accumulative scans ranging from 500 cm^−1^ to 4000 cm^−1^.

The surface morphologies of hydrogels were analyzed with scanning electron microscopy (EVO LS1503040702, Zeiss, Oberkohen, Batenwerburg, Germany). 

Preparation of hydrogel electron microscopy samples: The prepared hydrogel samples were lyophilized according to certain preparation methods and were not in expansion equilibrium. The lyophilized hydrogel samples were immersed in liquid nitrogen for 30 s, and the hydrogel samples were quickly broken off and relatively flat sections were taken upward to prepare the electron microscopy samples, sprayed with gold, and then subjected to scanning electron microscopy.

Rheological measurements were conducted on QAF and CAF hydrogels using a TA rheometer (DHR-1, Newcastle, DE, USA). To preparation a gel sample for the rheometer, different components of QAF hydrogel were quickly mixed, 0.1 mL was removed and dropped onto the platform of rheometer, the splint was dropped and the hydrogel sheet was formed. The hydrogels were prepared as cylindrical discs with a thickness of 0.2 mm and a diameter of 40 mm. Time sweep measurements were performed at a constant frequency of 1 Hz and a strain of 1% at room temperature to evaluate the storage modulus (G′) and loss modulus (G″) of the hydrogels. The range of the sweep time was set from 0 s to 300 s. The sample stage temperature was maintained at a constant 37 °C throughout the testing process.

The freeze-dried hydrogels were processed into similar shapes at room temperature and then weighed. The uptake properties of the hydrogels were measured following immersion in PBS (pH 7.4) until hydrogels reached a balance of uptake. The fully swelled hydrogels were weighed after absorbing the moisture on their surfaces by filter papers. The uptake rate of hydrogels was calculated using Equation (1):

(1) $$\mathrm{Uptake}\;\mathrm{rate}\;\left(\%\right)=\frac{m_{t}-m_{0}}{m_{0}}\times 100\%$$

where m_0_ and m_t_ are the initial weight and the swelled weight of hydrogels, respectively. The test was performed in triplicate under the same condition.

### 2.5. Self-Healing Performances of QAF Hydrogels

The hydrogels were split into two pieces, and the two parts were made to come in contact with each at 37 °C without external stimulation. Then, the hydrogels were lifted to determine whether they could support the weight of themselves.

Cylindrical discs with a thickness of 0.2 mm and a diameter of 40 mm were prepared from the hydrogels. The critical strain was measured using a TA rheometer by sweeping the strain amplitude with the strain range from 1% to 500%. Alternate step strain sweep measurements were performed to detect the self-healing performance at the fixed angular frequency of 1 Hz. Amplitude oscillation strains were switched from small strain (γ = 1%, 120 s for each interval) to large strain (γ = 200%, 120 s for each interval).

### 2.6. Antibacterial Activities

The antibacterial behavior of QAF and CAF hydrogels was evaluated using the plate count method with *S. aureus* and *E. coli* as model bacteria. In this study, freeze-dried hydrogel samples with a diameter of 1 cm and a thickness of 4 mm were incubated with a bacterial suspension containing 10^6^ CFU/mL for 24 h in a 24-well plate. Blank wells were used as a control. Subsequently, 10 μL of co-cultured bacterial solution was plated on LB agar and incubated at 37 °C for 24 h. The experiment was repeated three times under the same conditions, and the reduction rates of bacteria were calculated by counting the numbers of CFUs on agar plates using Equation (2):

(2) $$\mathrm{Reduction}\;\mathrm{rate}\;\mathrm{of}\;\mathrm{bacteria}\;\left(\%\right)=\frac{q_{\mathrm{control}}-q_{\mathrm{sample}}}{q_{\mathrm{sample}}}\times 100\%$$

The hydrogels were treated in the same manner as described above and cultured with 10^6^ CFU/mL bacterial suspension for 24 h. The samples were stained with fluorescein diacetate (FDA) and propidium iodide (PI) for 30 min and centrifuged. A fluorescence microscope (Leica, Wetzlar, Germany) was used to observe live (green) and dead (red) bacteria. The blank well was considered as a control.

The hydrogels were treated as described above and cultured with 10^6^ CFU/mL bacterial suspension for 24 h. After removing the supernatant, 2% (*v*/*v*) glutaraldehyde was added to fix the bacteria and the morphologies of bacteria were observed using SEM.

### 2.7. In Vitro Hemocompatibility and Cytocompatibility Evaluation

The viability of L929 fibroblasts in a culture experiment was measured to evaluate the cytotoxicity of the hydrogels using their extracts. The extracts were prepared by immersing the hydrogels in fresh DMEM medium at 37 °C for 24 h. Subsequently, three generations of L929 fibroblasts were inoculated on 96-well plate with a density of 8 × 10^3^ cells/well in DMEM medium supplemented with 10% FBS, which was incubated at 37 °C in a humidified atmosphere of 5% CO_2_ for 24 h. The extracts were then added to the wells to replace the original DMEM and CCK-8 was added to each well before incubating it at 37 °C for 1 h. Blank wells were used as controls, with their mediums refreshed. The OD value of the solution was measured at 450 nm. The test was performed in triplicate under the same conditions. The cell viability was calculated using Equation (3):

(3) $$\mathrm{Cell}\;\mathrm{viability}\;\left(\%\right)=\frac{{\mathrm{OD}}_{\mathrm{sample}}}{{\mathrm{OD}}_{\mathrm{control}}}\times 100\%$$

Erythrocytes were isolated from mouse blood by centrifugation (2500 × rpm, 4 °C, 10 min). To evaluate the hemocompatibility of hydrogels, 100 µL of hydrogel was mixed with 500 µL of erythrocyte stock and 500 µL of PBS, and incubated at 37 °C for 1 h. The mixed solution was centrifuged at 1000× rpm for 15 min and 100 µL supernatant of each group was added to a 96-well microplate. The OD values were read by a microplate reader at 545 nm. 0.1% Triton x-100 served as the positive control, while PBS buffer served as the negative control. The hemolysis rate was calculated using Equation (4):

(4) $$\mathrm{Hemolysis}\;\mathrm{rate}\;\left(\%\right)=\frac{{\mathrm{OD}}_{x}-{\mathrm{OD}}_{p}}{{\mathrm{OD}}_{t}-{\mathrm{OD}}_{p}}\times 100\%$$

where OD_x_, OD_p_, and OD_t_ were the OD values for the experimental group, PBS buffer negative control and 0.1% Triton x-100 positive control, respectively.

100 μL of mouse eyeball blood was added to the surface of prepared hydrogels, followed by the addition of 10 μL of 0.2 M CaCl_2_ solution, and the hydrogels were incubated at 37 °C for 10 min. The hydrogels were immersed in deionized water, and the absorbance was measured at 540 nm. The absorbance of whole blood in deionized water was used for reference. The test was performed in triplicate under the same conditions. The blood clotting index (BCI) of samples was calculated using Equation (5):

(5) $$\mathrm{BCI}\;\left(\%\right)=\frac{A_{\mathrm{sample}}}{A_{\mathrm{control}}}\times 100\%$$

For in vivo evaluation, a mouse was anesthetized and its liver was taken out by abdominal incision and placed on filter paper. Bleeding from the liver was induced using a needle, and hydrogel solution was immediately applied onto the bleeding site. Two minutes later, the weight of the filter paper with absorbed blood was measured and compared with a control group that received no treatment after pricking the liver.

### 2.8. Animal Studies

All animal experiments were approved by the Institutional Animal Care of Binzhou Medical University and animals were treated humanely during the entire process. Male mice (BALB-c, 8 weeks of age) were anaesthetized and full-thickness cutaneous defects (with a diameter of 0.8 cm) were created on the back. Wounds were treated with either QAF hydrogels, CAF hydrogels or athletic tape (control group). The wound healing of each mouse was recorded at 3, 7, 10 and 14 d. The wound healing rates were calculated using Equation (6):

(6) $$\mathrm{Wound}\;\mathrm{healing}\;\mathrm{rate}\;\left(\%\right)=\frac{A_{t}}{A_{0}}\times 100\%$$

where A_0_ is the initial area of wound and A_t_ is the area of wound at 3, 7, 10 and 14 day.

Further, tissues on wound sites were excised at the designated time point and processed for histological evaluation.

### 2.9. Statistical Analysis

All experimental data were analyzed using one-way analysis of variance followed by Bonferroni’s post-hoc test in GraphPad Prism v8.03 (GraphPad Software Inc., San Diego, CA, USA). The data were represented as means ± standard deviations (SDs) of measurements (* *p* < 0.05, ** *p* < 0.01, *** *p* < 0.001 and **** *p* < 0.0001). Each experiment was repeated three times.

## 3. Results and Discussion

### 3.1. Preparation and Structural Characterizations of Hydrogels

**Figure 1:**
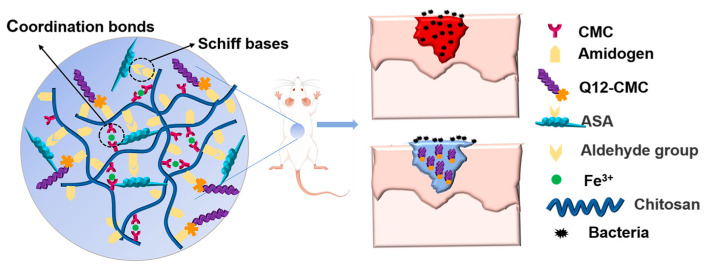
Schematic illustration of the preparation and application of QAF hydrogels.

**Figure 2:**
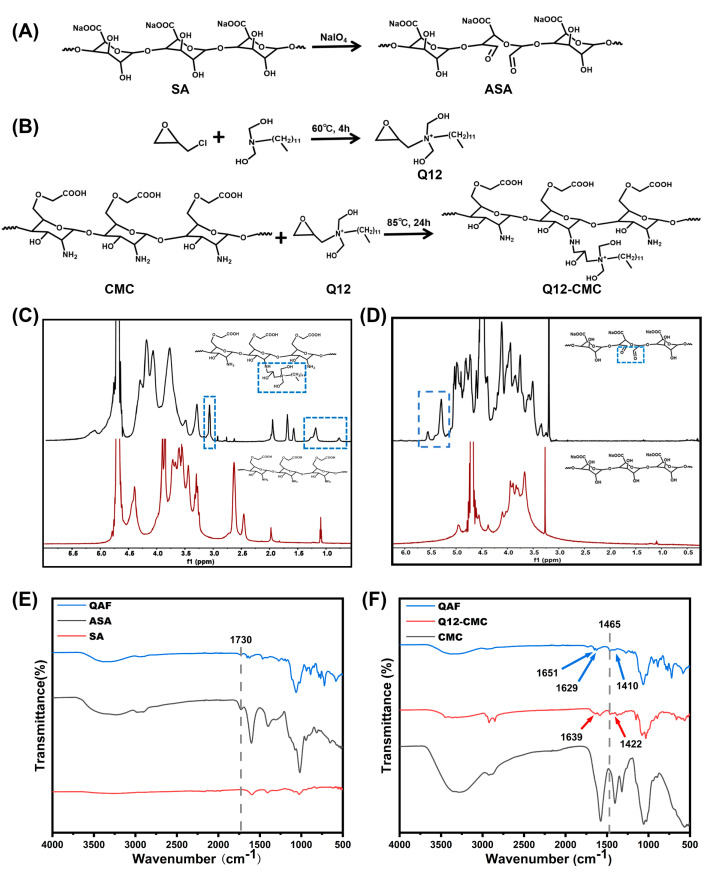
Preparation and structural characterizations of hydrogels. (**A**,**B**) Route of synthesis of ASA, Q12 and Q12-CMC; (**C**) 1H NMR spectrum of CMC and Q12-CMC (The dotted box indicates the NMR peak corresponding to the dodecyl quaternary ammonium salt); (**D**) 1H NMR spectrum of SA and ASA (The dotted box indicates the NMR peak corresponding to the aldehyde group); (**E**,**F**) FTIR spectra of SA, ASA, CMC, Q12-CMC and QAF hydrogels.

In view of the characteristics of wounds, double cross-linked hydrogels with excellent self-healing properties and antibacterial properties were designed (Figure 1). CS and SA, which were derived from marine polysaccharide, were chosen as the main materials of QAF hydrogels. ASA was obtained by oxidation of oxhydryl (adjacent hydroxyl groups) on the sodium alginate chain (Figure 2A). Antibacterial Q12 was introduced to CMC to prepare Q12-CMC (Figure 2B). QAF hydrogels were prepared by the coordination bonds between carboxymethyl in Q12-CMC and Fe^3+^, and Schiff bases between the amino groups in Q12-CMC and the aldehyde groups in ASA. The reaction process was characterized, and the results obtained were consistent with expectations. The ^1^H NMR spectra of both CMC and Q12-CMC are shown in Figure 2C. A comparison between the spectra revealed the presence of a new peak at 3.06 ppm in the ^1^H NMR spectrum of Q12-CMC, which was identified as the proton signal of N-CH_2_. Meanwhile, the successful introduction of long chain alkyl was confirmed by characteristic peaks at 1.19 and 0.77 ppm, corresponding to -CH_2_- and -CH_3_, respectively.

Figure 2E showed the FTIR spectra of SA and ASA. A new stretching vibration peak of aldehyde groups at 1730 cm^−1^ appeared in ASA, indicating aldehyde groups were successfully introduced into SA by periodate oxidation. The peak weakened greatly in QAF hydrogels, indicating the -CHO groups in ASA were consumed in Schiff base reactions. Figure 2F showed the FTIR spectra of CMC, Q12-CMC and QAF hydrogels. Compared with CMC, a new characteristic peak at 1465 cm^−1^ in Q12-CMC may be attributed to C-H stretching vibration of QAC. The formation of the Schiff bond consumes part of the aldehyde group and amino group, which can be seen from the infrared spectrum. The peak intensity at 1730 cm^−1^ in the QAF infrared spectrum is weakened. The QAF hydrogels exhibited a characteristic peak of imine bond at 1651 cm^−1^ in the FTIR spectra, indicating the occurrence of Schiff base reactions. In addition, it was worth noting that owing to the formation of coordination bonds, the -COO- peaks in the QAF hydrogels emerged at 1629 and 1410 cm^−1^, which were deviated from its normal position at 1639 and 1422 cm^−1^.

**Figure 3:**
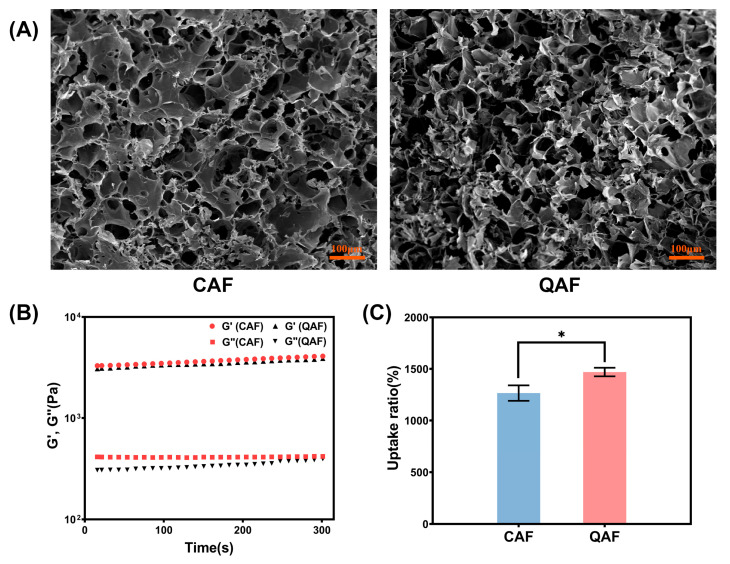
(**A**) SEM images, (**B**) rheological properties and (**C**) uptake ratios (*n* = 3) of QAF hydrogels and CAF hydrogels. * *p* < 0.05.

The morphologies of QAF hydrogels and CAF hydrogels were further observed by SEM. Figure 3A clearly illustrates that the hydrogels possessed a significant number of micro-porous structures. This can be attributed to the formation of dense and uniform networks through the coordination bonds and Schiff bases. The presence of these micro-pores enabled the hydrogels to maintain a moist environment, which is crucial for wound healing, while simultaneously ensuring that the dressing remained permeable [36,37].

To evaluate the rheological properties of the hydrogel, the storage modulus (G′) and loss modulus (G″) were measured as a function of time. The results, as depicted in Figure 3B, revealed that both samples had a higher G′ than G″, with G′ always being one order of magnitude greater than G″, which is consistent with the formation of hydrogels. Notably, there was no significant difference observed in the storage modulus (G′) between the QAF and CAF hydrogels, indicating that the introduction of long chain alkyl QAS had little effect on the rheological properties of QAF hydrogels.

The uptake capacity of hydrogels is a critical attribute for hydrogel-based wound dressings, as it contributes to the absorption of exudates and maintenance of a moist environment at the wound site [38]. This property can be characterized by the uptake ratio, which is determined by observing the hydrogel’s uptake behavior in PBS (pH 7.4) until a balance was reached. The results, presented in Figure 3C, revealed that both QAF and CAF hydrogels exhibited good uptake abilities, reflecting their highly porous network structure, as confirmed by morphological characterization. Therefore, the free water could easily enter the porous network structure of hydrogels. It is worth noting that the uptake ratio of QAF hydrogels was slightly higher than that of CAF hydrogels. Such difference could be attributed to the fact that QAS changed the pore structure of the hydrogels, thus improving uptake capacities of QAF hydrogels.

### 3.2. Self-Healing Properties

**Figure 4:**
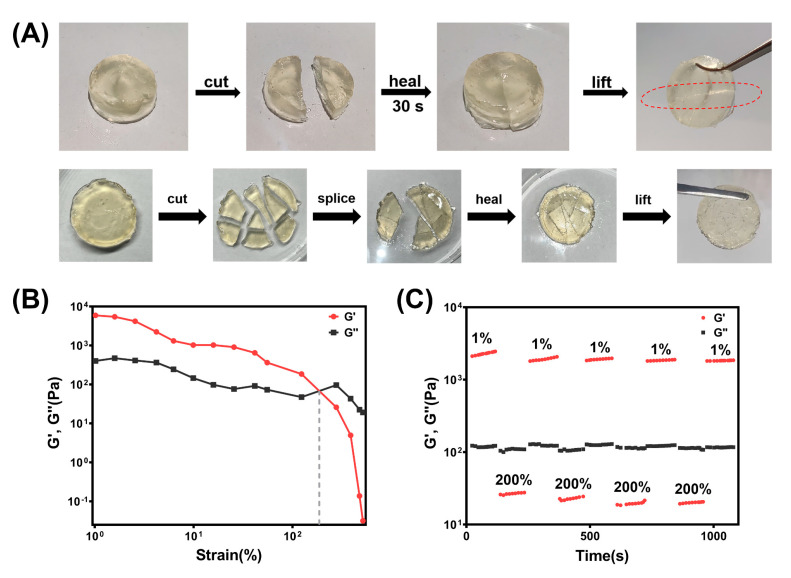
Self-healing abilities of the QAF hydrogels. (**A**) Macroscopic self-healing tests; (**B**) Strain amplitude sweep measurements (γ = 1–500%); (**C**) Alternate step strain sweep measurements with small strain (γ = 1%) to large strain (γ = 200%) with 120 s for every interval.

Hydrogel dressings are easily damaged by external mechanical forces, and traditional hydrogel dressings cannot repair themselves. The self-healing hydrogels can self-heal upon breakage, thus prolonging the service life and preventing wound infections [39]. The self-healing properties of QAF hydrogels were demonstrated through macroscopic observation, as exhibited in Figure 4A. The separated hydrogels autonomously merged into a circular cylinder again without any external stimulus within 30 s at body temperature, which could be lifted to support its own weight without rupture. The self-healing abilities of the proposed hydrogels could be attributed to dynamic coordination bonds and Schiff bases.

In addition, because the crosslinking mode and crosslinking group of CAF and QAF are basically the same, QAF is only partially grafted with long-chain quaternary ammonium salt, which has little effect on self-healing properties, so it can be considered that the self-healing properties of CAF and QAF are basically the same.

In order to quantify the self-healing properties of QAF hydrogels, a further rheological recovery test was conducted. The intersection point between the storage modulus (G′) and loss modulus (G″) curves occurred at approximately 185% strain, which was identified as the critical point (Figure 4B). As the strain increased beyond this point, G′ decreased dramatically and became lower than G″, indicating the collapse of the hydrogel networks. To assess the rheological recovery behavior, alternate step strain sweep measurements were performed. The result indicated that QAF hydrogels showed greater G′ than G″ when placed at 37 °C under 1% strain for 120 s (Figure 4C). When hydrogels were subjected to a strain of 200% for 120 s, G″ became greater than G′, which indicated the collapse of hydrogel structures [40]. It is noteworthy that that G′ and G″ recovered to the initial values once the strain returned to 1%. The collapse and recovery of hydrogel networks could be alternatively repeated several times, indicating the excellent self-healing capacities of QAF hydrogels.

### 3.3. Antibacterial Activities

**Figure 5:**
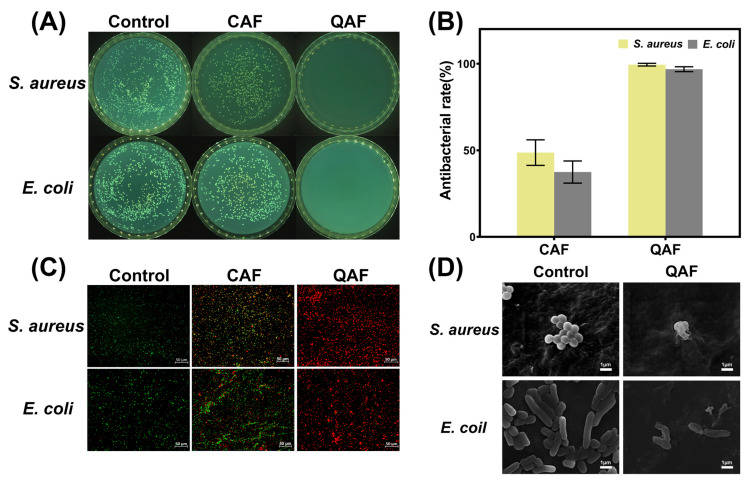
Antibacterial properties of hydrogels. (**A**) The counter board pictures of *S. aureus* and *E. coli* growth on QAF and CAF hydrogels; (**B**) Antibacterial rates of QAF and CAF hydrogels (*n* = 3); (**C**) FDA/PI fluorescence staining images of *S. aureus* and *E. coli* cultured with extracts of QAF and CAF hydrogels for 12 h. (**D**) SEM images of *S. aureus* and *E. coli* after contact with QAF hydrogels for 12 h.

Antibacterial properties are highly desired for wound dressings since they can significantly reduce the incidence of infections. In this study, we evaluated the antibacterial activities of hydrogels containing QAF and CAF against *S. aureus* and *E. coli*. The counter board pictures of respective bacterial growth on different hydrogels are presented in Figure 5A. Compared with CAF hydrogels, the suspension with QAF hydrogels was clearer, suggesting that QAF hydrogels had strong bactericidal activities. The quantitative analysis presented in Figure 5B revealed that QAF hydrogels were effective in inhibiting more than 98% of *S. aureus* and *E. coli*, while CAF hydrogels showed only a 50% reduction in the same strains. The superior antibacterial properties of QAF hydrogels were further validated by fluorescence staining, as shown in Figure 5C. Additionally, morphological changes in *S. aureus* and *E. coli* were examined to explore the antibacterial mechanism. The results showed that after contact with QAF hydrogels, the cell wall and membrane of bacteria were destroyed, resulting in a significant alteration in their shape (Figure 5D). The synergistic effect of quaternary amine groups with 12 carbon alkyl chains, Fe^3+^ and free amino groups of QAF hydrogels appeared to be responsible for targeting the negatively charged lipid membranes of microorganisms through electrostatic interaction. This mechanism of action resulted in the lysis and death of bacteria [41]. It was worth noting that the tolerance of *S. aureus* and *E. coli* to QAF hydrogels differed slightly, possibly due to the differences in their cytoderm and cytomembrane structure between gram-positive and gram-negative strains [42].

### 3.4. In Vitro Hemocompatibility and Cytocompatibility Evaluation

The incorporation of QAS into hydrogels can result in strong antibacterial activities. However, concerns have been raised regarding their potential adverse effects on hemocompatibility and cytocompatibility. Specifically, QAS moieties have been reported to interact with proteins and red blood cells (RBCs) in damaged tissue [43]. This interaction is facilitated by the electrostatic attraction between the positively charged QAS functional groups and the negatively charged RBCs. It has been shown that this interaction can result in lysis of RBCs due to changes in cell membrane molecular organization and permeability [44]. To mitigate the adverse effect of embedded QAS moieties in dressings membranes, CMC and ASA were incorporated into hydrogel wound dressings. We further evaluated the in vitro hemocompatibility and cytocompatibility of proposed hydrogels.

**Figure 6:**
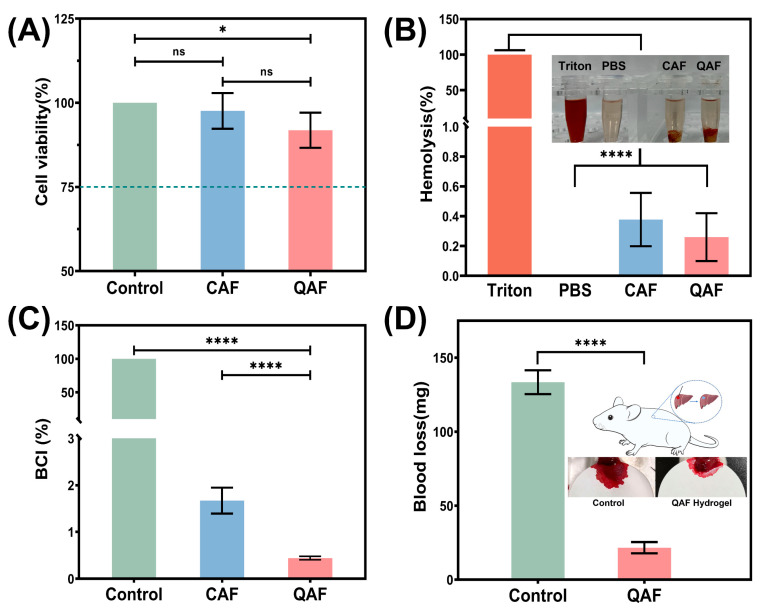
In vitro hemocompatibility evaluation. (**A**) Cell viabilities of L929 fibroblast cells (*n* = 3); (**B**) Hemolysis performances of hydrogels (*n* = 3); (**C**) The BCI values of whole blood clotting evaluation (*n* = 3); (**D**) Hemostatic performances of QAF hydrogels in mouse liver hemorrhage model (The control mouse received no treatment, *n* = 3). ns > 0.05, * *p* < 0.05, **** *p* < 0.0001.

To investigate the potential cytotoxic effects of QAS groups, the cell viabilities were examined by the leaching liquid assay using L929 fibroblast cells. As shown in Figure 6A, cell viabilities decreased upon the incorporation of QAS groups, indicating that the presence of positively charged QAS moieties was accountable for the reduction in cell viabilities [45]. Fortunately, the introduction of CMC and ASA possibly masked cationic charges of QAS and improved the surface hydrophilicity of the QAF hydrogels, resulting in cell viabilities within the acceptable limits [46].

The blood compatibility of QAF hydrogels was evaluated through in vitro hemolysis tests, and the results are shown in Figure 6B. After a one-hour incubation in a simulated physiological environment, QAF and CAF hydrogels were slightly light yellow in color, resembling the color of PBS, whereas the positive control group was bright red. Quantitative analysis revealed that the hemolysis ratio of QAF hydrogels was slightly higher than that of CAF hydrogels. This phenomenon was consistent with earlier report that red blood cells (RBC) were vulnerable to rupture in contact with positive charges of QAS moieties [44]. Nevertheless, the hemolysis ratio of QAF hydrogels was 0.26%, suggesting that the QAF hydrogels had minimal hemolytic effects in vitro and that the RBCs retained their function and integrity. It is well known that surface hydrophilicity is a dominant parameter influencing the hemolysis of biomaterials. Changing surface hydrophility by CMC and ASA might be crucial for improving cytocompatibility of QAF hydrogels.

BCI is a relative parameter to reflect the aggregation abilities of red blood cells, and a lower BCI value indicates better hemostatic properties of hydrogels. As shown in Figure 6C, the BCI value of QAF hydrogels was 0.44%, indicating that QAF hydrogels possessed superior hemostatic properties. The hemostatic performance of QAF hydrogels was further evaluated in a mouse liver hemorrhage model. The results depicted in Figure 6D demonstrated a significant reduction in blood loss (21.67 ± 3.76 mg) compared to the blank group (133.40 ± 8.06 mg). QAF hydrogels achieved their hemostatic effects by forming a stable gel network with good tissue adhesion abilities such that they could tightly adhere to the wound and provide a physical barrier to promote blood clotting. Furthermore, the positively charged quaternary ammonium groups and Fe^3+^ may induce hemagglutination by attracting negatively charged residues on RBC membranes. These groups may also lead to the adsorption of fibrinogen and plasma proteins, as well as the enhancement of platelet aggregation [47].

### 3.5. In Vivo Wound Healing Performance of Hydrogels

**Figure 7:**
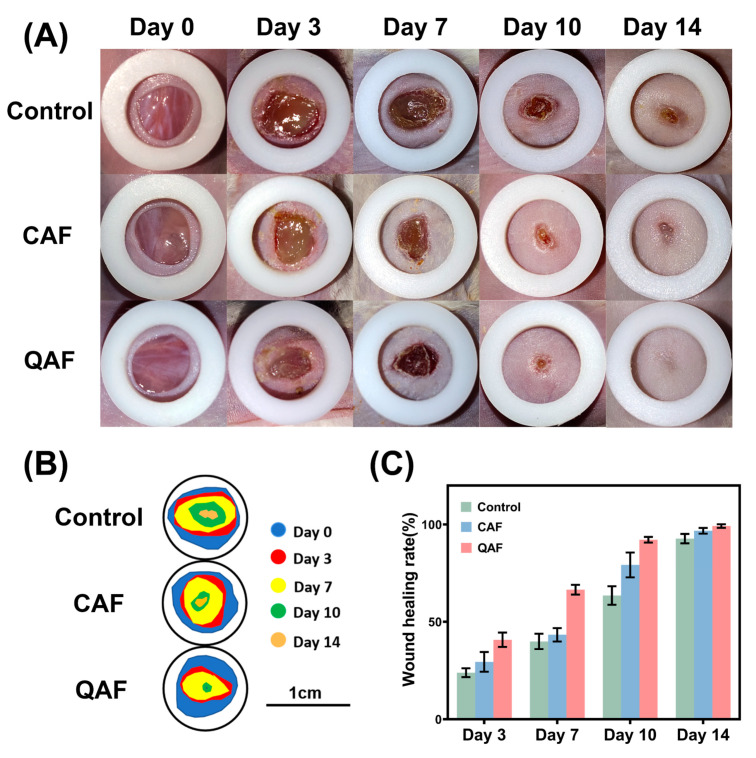
(**A**) Images of the wound healing sites on days 0, 3, 7, 10 and 14; (**B**) Traces of wound-bed closure on days 0, 3, 7, 10 and 14. (**C**) Quantitative analysis of wound healing rates according to Figure 7 (**A**) (*n* = 3).

Hydrogels for wound healing efficiency were assessed in a full-thickness skin defect model. Figure 7 shows wound healing performance after treatment with athletic tape (control group), QAF and CAF groups on days 0, 3, 7, 10 and 14, respectively. After 14 days, wound contraction was observed in all three groups. On day 3, the control group still showed some pus at the wound site, while the QAF hydrogel group had already started to heal. On day 7 post-surgery, the QAF hydrogel group exhibited a 66.45% wound healing rate, which was better than that of control groups (39.95%) and CAF groups (43.34%). This difference was attributed to the superior antibacterial properties of hydrogels, which facilitated early scab healing. Moreover, only the wound treated with QAF hydrogels exhibited complete closure on day 14, whereas the other groups still presented unhealed wounds, indicating the exceptional wound healing effects of QAF hydrogels.

**Figure 8:**
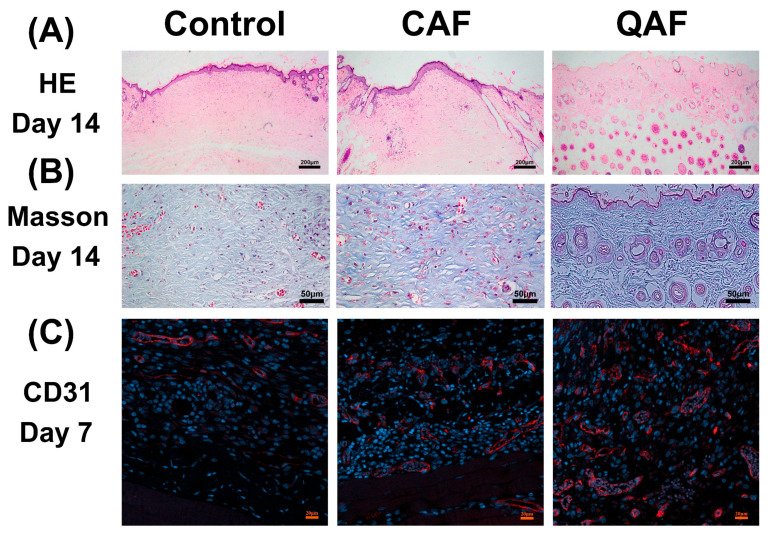
(**A**) HE staining of wound sections at day 14; (**B**) Masson’s staining of wound sections at day 14; (**C**) Immunofluorescence staining of CD31 structures at day 7.

Moreover, the effects of QAF hydrogels on wound healing were evaluated by HE staining to examine the inflammatory response and cell proliferation. The incision sites treated with QAF hydrogels exhibited superior healing properties without severe opening, as evidenced by the HE staining images presented in Figure 8A. Specifically, the QAF group had fewer inflammatory cells and more newly formed blood vessels and hair follicles compared to the control groups. To further investigate the biological mechanism and efficacy of QAF hydrogels in promoting wound repair, Masson’s staining and CD31 immunostaining were employed to evaluate the deposition of collagen and neovascularization, respectively. As shown in Figure 8B, the QAF group showed the highest collagen deposition compared to the other groups, indicating that QAF hydrogels could promote extracellular matrix deposition at the wound site and accelerate the healing process. Additionally, the immunofluorescence staining of CD31 in Figure 8C revealed significantly higher expression of CD31 protein in the QAF group compared to other groups, suggesting that QAF hydrogels could enhance angiogenesis and promote wound healing.

## 4. Conclusions

In this study, a dual-cross-linking self-healing hydrogel with efficient antibacterial properties using Q12-modified CMC, aldehyde group-modified SA, and Fe^3+^ via Schiff base and coordination bond was designed and easily fabricated. The dual-dynamic-bond cross-linked structure provided hydrogels with excellent self-healing capacities and uptake capacities. Furthermore, the addition of long chain alkyl QAS imparted potent antibacterial properties against *E. coli* and *S. aureus*. In vitro hemocompatibility and cytocompatibility tests demonstrated that the proposed hydrogels had good compatibility. The efficacy of the hydrogels in treating bacterial infections and accelerating wound healing was confirmed in a mouse full-thickness skin defect model. Therefore, this study offers a promising avenue for developing self-healing hydrogels with superior antibacterial properties for facilitating skin regeneration and wound healing. Furthermore, the hydrogel of this wound dressing type can be applied to bacterially infected wounds [48], burns [49] and repair of skeletal muscle tissue in the future [50]. The further optimization of products to make them more suitable for industrial development is a new direction to promote research and application.

## Data Availability

Not applicable.
